# Invasive Multidrug-Resistant *emm*93.0 *Streptococcus pyogenes* Strain Harboring a Novel Genomic Island, Israel, 2017–2019

**DOI:** 10.3201/eid2801.210733

**Published:** 2022-01

**Authors:** Merav Ron, Tal Brosh-Nissimov, Zinaida Korenman, Orit Treygerman, Orli Sagi, Lea Valinsky, Assaf Rokney

**Affiliations:** Ministry of Health, Jerusalem, Israel (M. Ron, Z. Korenman, L. Valinsky, A. Rokney);; Assuta Ashdod University Hospital, Ashdod, Israel (T. Brosh-Nissimov);; Ben Gurion University in the Negev, Beer Sheba, Israel (T. Brosh-Nissimov);; Meuhedet Health Services, Lod, Israel (O. Treygerman);; Soroka University Medical Center, Beer-Sheva, Israel (O. Sagi)

## Abstract

Invasive group A *Streptococcus* (iGAS) infections have increased in Israel since 2016 as successful lineages have emerged. We report the emergence and outbreak of a multidrug-resistant *S. pyogene*s *emm*93.0, sequence type 10, among iGAS infections in Israel since 2017. This type has been observed very rarely in other countries. During this period, *emm*93.0 was the cause of 116 infections in Israel and became the leading type during 2018. Most of the infections were from bacteremia (75%), and most patients were male (76%). We observed infections across Israel, mainly in adults. Of note, we observed multidrug resistance for clindamycin, tetracycline, and trimethoprim/sulfamethoxazole. Whole-genome sequencing confirmed clonality among geographically disseminated isolates. The local *emm*93.0 sequence type 10 clone contained a novel genomic island harboring the resistance genes *lsa(E)*, *lnu(B),* and *ant (6)-Ia aph(3′)-III*. Further phenotypic and genomic studies are required to determine the prevalence of this resistance element in other iGAS types.

*Streptococcus pyogenes* is a major cause of community-acquired and nosocomial infections linked with illness and death worldwide (*1*,*2*). Group A *Streptococcus* (GAS) species cause a variety of infections, including pharyngitis, skin and soft tissue infections (SSTI), severe invasive infections, bacteremia, and toxic shock syndrome (*3*, *4*). Acquisition of GAS is mainly attributed to person-to-person transmission by respiratory droplets or skin contact; in addition, inoculated food can spread the infection, resulting in outbreaks (*5*,*6*).

Surveillance programs and prevention guidelines focus on systemic GAS infection, which is defined as a statutory notifiable disease in many countries (*5*). Active laboratory-based surveillance based on molecular characterization of invasive isolates is essential for outbreak detection and public health response. However, laboratory surveillance data indicate that most invasive cases in industrialized countries occur sporadically and are not part of outbreak clusters (*5*). In Israel, iGAS is a notifiable disease; all invasive isolates are analyzed at the national reference laboratory at the Ministry of Health (Jerusalem, Israel).

The *emm* typing scheme is a primary tool for surveillance, outbreak detection, and for the study of the population structure on the basis of the sequencing of the *emm* gene (*7*). Specific *emm* types and M proteins (M1, M3) have been linked with invasive infections. Several studies show that *emm* types are significantly more diverse in developing countries than in developed countries (*5*).

Susceptibility testing is essential for successful outbreak control. Although β-lactams are the preferred antimicrobial drug treatment for GAS infections, macrolides, lincosamides, and streptogramins are useful for treating patients with β-lactam allergy, and for overcoming treatment failure in patients treated with penicillin. Recommendations for treating severe invasive cases include adding clindamycin or linezolid, which suppress toxin production (*8*,*9*). Several reports highlight the emergence of successful clones associated with acquired antimicrobial resistance (*10*–*12*). Clindamycin resistance is rarely reported and is associated with specific lineages (*13*,*14*).

We describe the epidemiology of invasive GAS (iGAS) in Israel during 2014–2019. We report the emergence and ongoing outbreak of a multidrug-resistant (MDR) *S. pyogenes emm*93.0 that caused 116 iGAS cases during 2016–2019 in Israel. The unique epidemiologic dynamics of the outbreak clone, as well as in-depth whole-genome sequence analysis, were the focus of our investigation.

## Materials and Methods

### Strain Typing and Susceptibility Testing

In Israel, all *S. pyogenes* strains isolated from normally sterile sites are referred to the national *Streptococcus* reference center as part of routine surveillance. All isolates are cultured on blood agar base plates (HyLabs, https://www.hylabs.co.il) or in Todd-Hewitt broth at 37°C. We conducted a survey of 66 noninvasive GAS strains from throat and ear samples received from 3 medical centers to detect carriage of *emm*93.0. We subjected all *S. pyogenes* isolates to Lancefield grouping (*15*) and to *emm* typing in accordance with the guidelines stated by the US Centers for Disease Control and Prevention (CDC; https://www2.cdc.gov/vaccines/biotech/strepblast.asp).

We determined the antibiotic susceptibility of *emm*93.0 strains by broth microdilution using Sensititer (TREK Diagnostic Systems, https://www.trekds.com) with STP6F antimicrobial susceptibility test (AST) plates containing 20 antimicrobial drugs, according to the manufacturer’s instructions. We used Sensititre Vizion (Thermo Fisher, https://www.thermofisher.com) for manual reading of growth. We interpreted MIC according to Clinical and Laboratory Standards Institute (CLSI) 2019 guidelines except for trimethoprim/sulfamethoxazole, for which CLSI breakpoints were unavailable; therefore, we used the epidemiologic cutoff value, 0.5 μg/mL, from the European Committee on Antimicrobial Susceptibility Testing (https://www.eucast.org/clinical_breakpoints).

### Whole-Genome Sequencing Analysis

Of 116 *emm*93.0 strains, we subjected 26 (22.4%), representing different isolation dates and geographic locations, to whole-genome sequencing (Appendix 1 Table 2). We performed DNA extraction using the QIAsymphony SP system and the QIAsymphony DNA mini kit (QIAGEN, https://www.qiagen.com) according to the manufacturer’s recommendations. We lysed culture pellets in a 180 µL enzymatic lysis buffer (20 mM Tris-Cl pH 8.0 (VWR Amresco, https://us.vwr.com), 2 mM sodium EDTA (VWR Amresco), 1.2% Triton X-100 (Sigma-Aldrich, https://www.sigmaaldrich.com) and lysozyme 20 mg/mL (Sigma-Aldrich). We incubated cell suspension for 30 min at 37°C, then treated with proteinase K and buffer AL (QIAGEN). We subjected silica beads in lysing matrix B bulk (MP Biomedicals, https://www.mpbio.com), in a volume equivalent to 150 μL, to the TissueLyser II (QIAGEN) for 2 cycles of 30 Hz for 30 s. We transferred supernatants to the QIAsymphony SP system. 

We sequenced Nextera XT DNA Libraries (Illumina, https://www.illumina.com) on MiSeq (Illumina), using a read length of 250 bp paired-end at >100× coverage. We analyzed reads by the BioNumerics version 7.6.3 (Applied Maths, https://www.applied-maths.com). We generated de novo assemblies using SPAdes version 3.7.1 (https://github.com/ablab/spades/tree/spades_3.7.1). To determine clonality of the outbreak strains, we mapped reads (Appendix 1 Table 2) against a reference strain (IST003) using the bowtie algorithm. We used the BioNumerics Gene Extraction tool (Applied Maths) to search the assemblies for sequences of antimicrobial resistance genes and virulence factors, in accordance with published gene sequences (Appendix 1 Table 3) (*16*). To identify the genetic basis for antimicrobial resistance, we analyzed the genomes using the Pathosystems Resource Integration Center (PATRIC) version 3.6.6 (*17*). We obtained visualizations for comparison of IST001, IST003, and GAS2887HUB (Sequence Read Archive [SRA] accession no. ERR2880947) putative prophage insertion site using the Easyfig program (*18*). We used Fisher exact test for statistical analysis and comparison of categorical variables between groups.

## Results

### Epidemiology of iGAS

**Figure 1:**
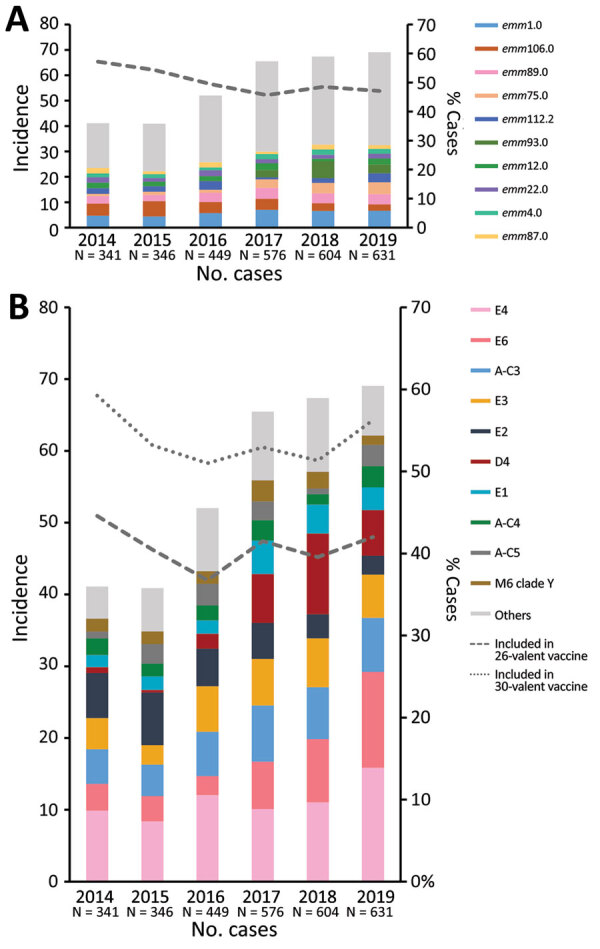
Incidence of *emm* types and clusters among 2,947 iGAS cases in Israel during 2014–2019 and potential vaccine coverage. A) Ten most common *emm* types, by incidence per 100,000 population for each year. Each color bar section represents 1 of the top 10 *emm* types; gray bar sections represent all other *emm* types. Dashed line represents the percentage of top 10 *emm* types from total cases each year. B) Ten most common *emm* clusters, by incidence per 100,000 population for each year. All *emm* types were assigned to *emm* clusters. Each color bar section represents a top 10 *emm* cluster; gray bar sections represent other *emm* clusters. Dashed line represents the percentage of potential coverage of 26-valent vaccine for each year; dotted line represents the percentage of potential coverage of 30-valent vaccine for each year. iGAS, invasive group A *Streptococcus*.

During 2014–2019, a total of 2,947 iGAS isolates were analyzed at the *Streptococcus* reference laboratory (Figure 1, panel A). The most common source of iGAS was blood (51.3%), followed by wounds (21.7%). The incidence of iGAS was 40–70 cases/100,000 population/year. We observed an increasing trend in incidence during 2016–2019 (Figure 1, panel A). A total of 180 *emm* types were identified; the 10 leading types were *emm*1.0 (n = 307, 10.4%), *emm*106.0 (n = 215, 7.3%), *emm*89.0 (n = 189, 6.4%), *emm*75.0 (n = 136, 4.6%), *emm*112.2 (n = 123, 4.2%), *emm*93.0 (n = 116, 3.9%), *emm*12.0 (n = 104, 3.5%), *emm*22.0 (n = 102, 3.5%), *emm*4.0 (n = 90, 3.0%), and *emm*87.0 (n = 84, 2.8%). The predominant type in Israel was the globally reported *emm*1.0 that caused 9.7%–11.4% of iGAS cases per year; it ranked first or second in each year (*19*). The top 10 *emm* types of each year accounted for 52%–60% of total iGAS cases (Appendix 1 Table 1). Among the 10 leading *emm* types, some remained stable throughout the studied period, with few exceptions (Figure 1; Appendix 1 Table 1). The 180 *emm* types identified during 2014–2019 were clustered into 38 *emm* acceptable clusters (*20*). The top 10 *emm* clusters accounted for >86% of total cases; they were E4 (N = 590, 20%), E6 (N = 343, 11.6%), A-C3 (N = 333, 11.3%), E3 (N = 287, 9.7%), E2 (E = 257, 8.7%), D4 (E = 247, 8.4%), E1 (N = 152, 5.2%), A-C4 (N = 117, 4%), A-C5 (N = 114, 3.9%), and M6 clade Y (N = 104, 3.5%) (Figure 1, panel B). The largest *emm* cluster, E4, peaked in 2019. It represented <20% of iGAS cases, and included mainly types *emm*89.0, *emm*112.2, and *emm*22.0. Cluster D4 was rarely detected during 2014–2016 (28 cases), but peaked in 2017–2019 (219 cases). The predominant types among cluster D4 were *emm*93.0 (47%), *emm*53.3 (24.7%), and *emm*33.0 (20.2%).

The potential coverage of multivalent vaccines was low in Israel compared with other industrialized countries (*21*). The yearly predicted coverage of the 26-valent *S. pyogenes* vaccine was 36.7%–44.6% of the invasive strains, and 40.7% for the entire period. The yearly predicted coverage of the 30-valent *S. pyogenes* vaccine was 51%–59.2% of invasive strains, and 53.7% for the entire period (Figure 1, panel B).

**Figure 2:**
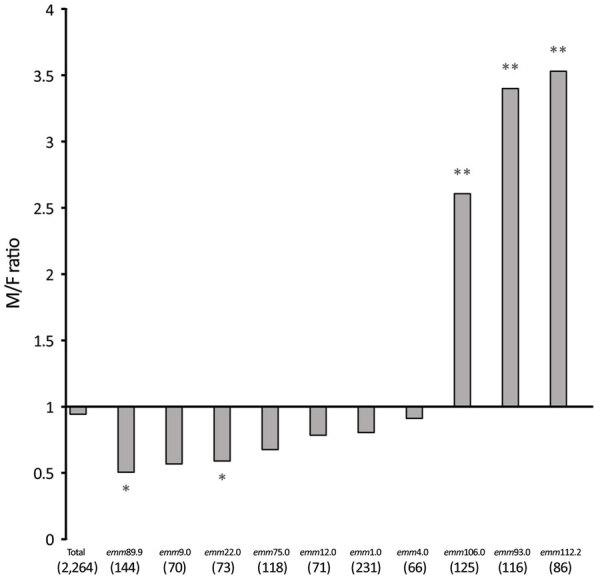
Ratio of male to female case-patients with invasive group A *Streptococcus* (N = 2,263) for selected *emm* types in Israel, 2016–2019. Asterisks (*) indicate significant results (p<0.05). Double asterisks (**) indicate significant results with Fisher exact test statistic value <0.00001.

**Figure 3:**
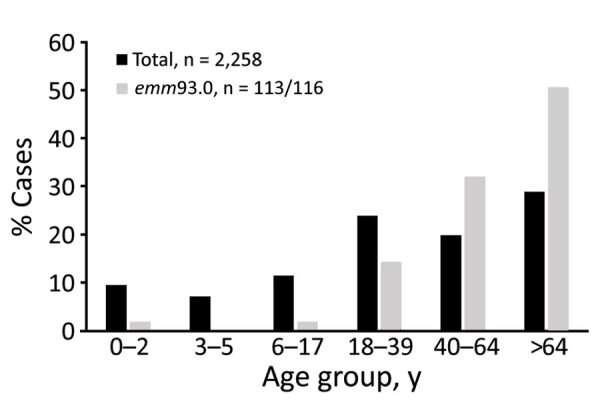
Age distribution among total invasive group A *Streptococcus* case-patients (N = 2,258) and *emm*93.0 case-patients (N = 116,of which 113 were of known age) Israel, 2016–2019.

During 2016–2019, a total of 2,263 iGAS cases were distributed almost evenly between sexes (48.2% male, 51.8% female). However, differences in sex of patients were noticeable in several *emm* types (Figure 2). Most patients (77%) with *emm*93.0 were male; *emm*106.0 and *emm*112.2 had significantly higher incidence (p<0.05) in male patients (Figure 2). In contrast, *emm*89.0 and *emm*9.0 were significantly more prevalent (p<0.05) in female patients (Figure 2). Most iGAS cases (72.2%) were reported in adults >17 years of age, and the most affected age group was >64 years of age (28.7%) (Figure 3). 

### *emm*93.0 Emergence and Outbreak

**Figure 4:**
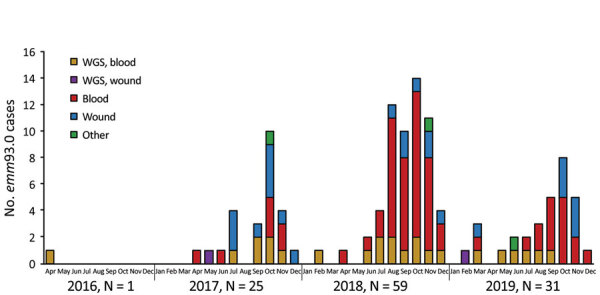
Epidemiologic curve of invasive group A *Streptococcus* *emm*93.0 type cases, Israel, April 2016–December 2019. Bar color indicates specimen source and whether the strains in the sample were analyzed by WGS. Other category includes sterile body fluid or vaginal swab specimen. WGS, whole-genome sequencing.

Our surveillance data highlight the emergence of *emm*93.0 during 2016, followed by an ongoing outbreak across Israel of this rarely reported type. After a single case in April 2016, *emm*93.0 emerged during 2017 and caused 4.3% of cases, 9.8% of cases in 2018, and 4.9% of cases in 2019. A total of 116 *emm*93.0 invasive GAS cases were diagnosed during April 2016–December 2019; cases peaked in October in 2017–2019 (Figure 4). The isolates were recovered mainly from blood (76% of total cases) and wound specimens. Thirteen (52%) of the isolates were from blood and 11 (44%) from wounds in 2017. In 2018, bacteremia cases yielded 51 (86%) isolates (Figure 4). Of note, in a survey of 66 throat and ear samples from the community, *emm*93.0 was not detected among noninvasive cases (Appendix 1 Table 4). Most emm93.0 cases were among adult patients from age groups 39–64 years (31%) and >64 years of age (51%), versus 20% of total iGAS cases from patients 39–64 years of age and 29% from those >64 years of age (Figure 3).

**Figure 5:**
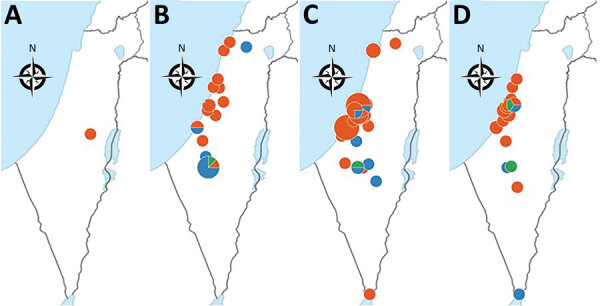
Demographic dispersal of invasive group A *Streptococcus* (iGAS) *emm*93.0 type cases by year, Israel. Colors indicate specimen source; other indicate sterile bodily fluids. Dot size is proportional to the number of cases. A) 2016, 1 case *of emm*93.0 B) 2017, 24/25 *emm*93.0 cases. C) 2018, 54/59 *emm*93.0 cases. D) 2019, 27/31 *emm*93.0 type of 31 iGAS cases.

We noted the geographic distribution of 106/116 (91.4%) *emm*93.0 cases (Figure 5). The earliest reported case of *emm*93.0 in Israel was in a patient from Judea and Samaria district. During 2017–2019, cases were reported from 17 medical centers and disseminated across Israel. The outbreak clone (82% of total *emm*93.0 cases) was distributed mainly in 3/7 districts of Israel: South, Tel-Aviv, and Center (Figure 5).

### Antimicrobial Resistance

The outbreak clones were notably resistant to clindamycin (MIC >1 μg/mL), tetracycline (MIC >8 μg/mL), and trimethoprim/sulfamethoxazole (MIC >4 μg/mL). All outbreak isolates were susceptible to azithromycin, cefepime, cefotaxime, ceftriaxone, chloramphenicol, daptomycin, ertapenem, erythromycin, levofloxacin, linezolid, meropenem, moxifloxacin, penicillin, and vancomycin. The earliest-reported *emm*93.0 isolate identified in Israel, IST001, was resistant to tetracycline (MIC >8 μg/mL) and trimethoprim/sulfamethoxazole (MIC >4 μg/mL) but susceptible to clindamycin (MIC ≤0.12 μg/mL) and all other antimicrobial drugs tested.

### Whole-Genome Sequencing Analysis

We investigated a sample of 26 isolates (22.4%) by whole-genome sequencing to confirm clonality and characterize the emerging type (Appendix 1 Table 3). We used the first known isolate from the southern district of Israel (IST003) as a reference sequence. Sequence mapping confirmed clonality of the outbreak strains isolated during 2017–2019, identifying <11 single-nucleotide polymorphism (SNPs). SNPs were accumulated during the outbreak years: <4 SNPs in 2017, <6 SNPs in 2018, and <11 SNPs in 2019 compared with the reference strain. We determined that IST001, the first *emm*93.0 isolate isolated in 2016, was not part of the outbreak from its high number of SNPs (>350 SNPs). SNPs were distributed throughout the genome; we observed 5 areas with dense SNPs (Appendix 1 Table 5; Appendix 2 Figure 1), which rules out the option of a single recombination event. We performed BLAST (https://blast.ncbi.nlm.nih.gov/Blast.cgi) analysis to map and identify the genes located in these areas. Among those genes were genes coding for proteins with various functions such as metabolism and biosynthesis (LacA, LacB, GatA, GatB, GatC, FabK, CodY) and transport (PTS lactose transporters, ABC transporters, ECF transporters); proteins involved in DNA replication, recombination, transcription and translation (MutL, RecG, XRE family, MarR family, ribosomal proteins S9, L13); toxins (holin-like, type II toxin-antitoxin system); and several proteins with unknown functions (Appendix 1 Table 5).

**Figure 6:**
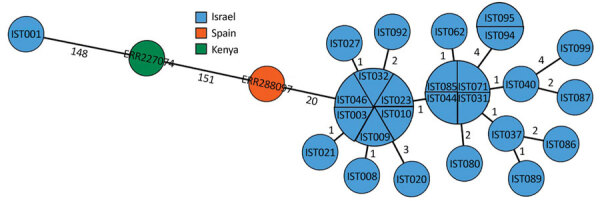
Minimum spanning tree of invasive group A *Streptococcus* *emm*93.0 type from Israel, 2016–2019, and global strains (Spain and Kenya). Tree is based on whole-genome multilocus sequence typing comparison of selected *emm*93.0 strains and global strains with logarithmic scaling. Node color represents the country origin of the sample. Nodes are labeled by sample number key for those from this outbreak; those from Spain and Kenya are labeled by Sequence Read Archive accession number (Appendix 1 Table 2). Numbers on branches indicate the number of allelic differences between those 2 strains.

We extracted sequences of 7 housekeeping genes (*gki, gtr, murI, mutS, recP, xpt, ypiL*) from the assemblies and submitted them to PubMLST (https://pubmlst.org) to determine the sequence type (ST) of the strains. All strains were ST10 and had the allelic profile 2-2-9-13-2-14-2. ST10 was reported in only 12 strains in pubMLST from tropical and subtropical regions, associated with 7 rare *emm* types, 93, 70, 80, 98, 108, 121, and 142. To further analyze the strains, we conducted whole-genome multilocus sequence typing (wgMLST) analysis using the BioNumerics *S. pyogenes* scheme (Applied Maths). Among the outbreak strains, we detected <9 allelic differences (Figure 6). We compared the Israel strains to globally available sequences of *emm*93.0 and detected 20 allelic differences from GAS2887HUB (SRA accession no. ERR2880947) a strain reported in Spain (*22*), and 153 allelic differences from an isolate from Kenya, K40810 (SRA accession no. ERR227074) (*23*).

### Antimicrobial-Resistance Genes and Genes Contributing To Virulence

**Figure 7:**
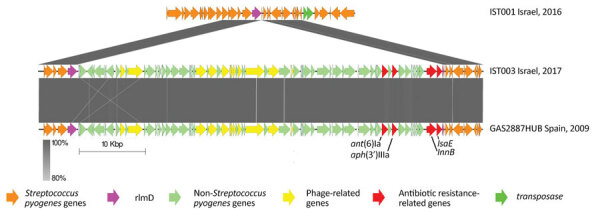
Schematic comparison of the integration site of the prophage carrying antimicrobial resistance genes for 2 invasive group A *Streptococcus* *emm*93.0 type strains from Israel and 1 from Spain. Arrows indicate gene arrangement in the presumed insertion site of the prophage, the *rlm*D gene (purple). The prophage contains gene sequences of antibiotic resistance related genes (*ant*(6)Ia, *aph*(3')IIIa*, lsa*E and *lnu*B), phage related genes and other non-*Streptococcus* genes. The gray regions indicate 80%–100% sequence identity.

We used PATRIC version 3.6.6 Specialty Genes Search tool to identify antimicrobial resistance genes in the genome of the resistant *S. pyogenes emm*93.0 isolate. We compared the search results of a resistant strain, IST003, to the sensitive strain, IST001; the genes found only in the resistant strain were *ant*(6)-I, *aph*(3′)-III *lnu*(B), and *lsa*(E). In addition, *tet*(M) was found in both strains. No amino acid replacement was found in PBP2x (*24*). To identify gene arrangement and their genomic environment, we searched gene sequences in de novo assembled genomes using BioNumerics platform tools. Using BLAST, we defined a genomic island 56,821-bp long, at IST003 positions 1427343–1484164. This region was identical to a genomic island harboring *lsa*(E), *lnu*(B), and a defective prophage in *S. pyogenes emm*93.0 strain, previously described in Spain (*22*). In the United States, the *lsa/lnu* type determinants are extremely rare in invasive GAS, but are not uncommon among invasive group B *Streptococcus* (*21*,*25*). The genomic island presumably integrated into the *rlm*D gene (Figure 7).

Among the SAg genes, we found *speB, speG, speM* and *smeZ* in all outbreak strains; we detected *slo* as well. The first *emm*93.0 clone, isolated in Israel (IST001) has a different pattern of genes: *speB, speC, speG, speH, speI, speM, smeZ, slo*, and *tetM* (Appendix 2 Figure 2; Appendix 1 Table 3).

## Discussion

We observed an increase in incidence of iGAS in Israel from 2014 to 2019 (40–70 cases/100,000 population). The iGAS population in Israel displayed high genetic diversity. Global data reflect limited diversity in industrialized countries, and higher diversity in developing countries. The consistently high iGAS diversity in Israel, an industrialized country, is noteworthy and concerning. The potential coverage of multivalent *emm*-type–specific vaccines is expected to be limited in Israel compared with vaccine coverage in Europe and the United States. In Israel the strain types included in the protein-based 26-valent vaccines cover 40.7% of cases and in the 30-valent vaccines, 53.7% of cases, compared with 79% coverage by the 26-valent vaccines and 91% of cases by the 30-valent vaccines in the United States (*26*).

The molecular surveillance of all iGAS cases reflects trends and fluctuations. The leading strain type in Israel during 2014–2019 was *emm*1.0, similar to global reports. Of note, *emm*93.0 has never been detected in the ongoing molecular surveillance of iGAS initiated in 2003. This type was not detected in a sample of noninvasive GAS, and the source and reservoir of this clone are currently unknown. Because cluster D was found to be associated with impetigo and skin infections (*27*,*28*), it could be related to skin carriage. In the United States, outbreak clusters were associated with increasingly more invasive GAS (*emm* types 49, 82, and 92) (*21*).

The *emm*93.0 type has been reported very rarely in other countries. In Spain, a single case of iGAS in 2009 was caused by an MDR *emm*93.0 isolate carrying a defective prophage (*22*). Most characterized *S. pyogenes* exotoxins, deoxyribonucleases, or DNases are carried by prophages, which contribute to its virulence, immune evasion, and bacterial DNA degradation (*29*). We compared the Israel outbreak’s WGS profile with the case in Spain and report a resemblance, with 20 allelic differences identified by wgMLST. These differences were scattered in different genomic regions and not likely due to a single recombination event. The reported resistance phenotype and genotype of the Spain isolate were identical to those of the outbreak cluster in Israel. The prevalence of this type in Spain is currently unknown. A study of GAS in New Caledonia reported 18 isolates of *emm*93 among 318 cases (5.6%) from 2012 (*30*); a single invasive case was reported, but subtype and antimicrobial susceptibility were not reported.

The emergence and outbreak of an MDR clone among iGAS cases is concerning in 2 ways. First, many patients with SSTI and a history of β-lactam allergy receive empiric treatment with clindamycin, but results of antimicrobial susceptibility testing are unknown for many of these patients because cultures from SSTIs are not commonly positive. Second, patients with invasive streptococcal infections and hemodynamic instability are commonly treated with clindamycin to inhibit toxin production. If iGAS clones with clindamycin resistance become prevalent, a different empiric approach might be considered, such as the use of linezolid.

The emerging *emm*93.0 type is not covered by the multivalent vaccines under development. The high genetic similarity of the Israel outbreak clone to a single case from Spain from 2009 may indicate an epidemiologic link and global transmission. Systematic molecular surveillance of iGAS is essential for detection of local and global emerging clones and for evidence-based vaccine development and distribution.

Appendix 1 Additional tables for study of invasive multidrug-resistant group A *Streptococcus* *pyogenes emm*93.0 type outbreak, Israel, 2017–2019.

Appendix 2 Additional figures for study of invasive multidrug-resistant group A *Streptococcus* *pyogenes emm*93.0 type outbreak, Israel, 2017–2019.

## References

[1] Ralph AP, Carapetis JR. Group a streptococcal diseases and their global burden. Curr Top Microbiol Immunol. 2013;368:1–27. 23242849 10.1007/82_2012_280

[2] Carapetis JR, Steer AC, Mulholland EK, Weber M. The global burden of group A streptococcal diseases. Lancet Infect Dis. 2005;5:685–94. 10.1016/S1473-3099(05)70267-X 16253886

[3] Barnett TC, Bowen AC, Carapetis JR. The fall and rise of Group A *Streptococcus* diseases. Epidemiol Infect. 2018;147:e4. 10.1017/S0950268818002285 30109840 PMC6518539

[4] Henningham A, Barnett TC, Maamary PG, Walker MJ. Pathogenesis of group A streptococcal infections. Discov Med. 2012;13:329–42. 22642914

[5] Efstratiou A, Lamagni T. Epidemiology of *Streptococcus pyogenes.* In: Ferretti JJ, Stevens DL, Fischetti VA, editors. *Streptococcus pyogenes:* basic biology to clinical manifestations. Oklahoma City (OK): University of Oklahoma Health Sciences Center; 2016. 26866208

[6] Kemble SK, Westbrook A, Lynfield R, Bogard A, Koktavy N, Gall K, et al. Foodborne outbreak of group a *streptococcus* pharyngitis associated with a high school dance team banquet—Minnesota, 2012. Clin Infect Dis. 2013;57:648–54. 10.1093/cid/cit359 23868521

[7] Smeesters PR, Botteaux A. The *emm*-cluster typing system. Methods Mol Biol. 2020;2136:25–31. 10.1007/978-1-0716-0467-0_3 32430811

[8] Diep BA, Equils O, Huang DB, Gladue R. Linezolid effects on bacterial toxin production and host immune response: review of the evidence. Curr Ther Res Clin Exp. 2012;73:86–102. 10.1016/j.curtheres.2012.04.002 24648596 PMC3954010

[9] Zimbelman J, Palmer A, Todd J. Improved outcome of clindamycin compared with beta-lactam antibiotic treatment for invasive *Streptococcus pyogenes* infection. Pediatr Infect Dis J. 1999;18:1096–100. 10.1097/00006454-199912000-00014 10608632

[10] Oppegaard O, Skrede S, Mylvaganam H, Kittang BR. Emerging threat of antimicrobial resistance in β-hemolytic streptococci. Front Microbiol. 2020;11:797. 10.3389/fmicb.2020.00797 32477287 PMC7242567

[11] Tsai WC, Shen CF, Lin YL, Shen FC, Tsai PJ, Wang SY, et al. Emergence of macrolide-resistant *Streptococcus pyogenes emm*12 in southern Taiwan from 2000 to 2019. J Microbiol Immunol Infect. 2020;•••:S1684-1182(20)30217-6; Epub ahead of print. 10.1016/j.jmii.2020.08.019 32994137

[12] Brouwer S, Barnett TC, Ly D, Kasper KJ, De Oliveira DMP, Rivera-Hernandez T, et al. Prophage exotoxins enhance colonization fitness in epidemic scarlet fever-causing *Streptococcus pyogenes.* Nat Commun. 2020;11:5018. 10.1038/s41467-020-18700-5 33024089 PMC7538557

[13] Pesola AK, Sihvonen R, Lindholm L, Pätäri-Sampo A. Clindamycin resistant *emm*33 *Streptococcus pyogenes* emerged among invasive infections in Helsinki metropolitan area, Finland, 2012 to 2013. Euro Surveill. 2015;20:21117. 10.2807/1560-7917.ES2015.20.18.21117 25990232

[14] Richter SS, Heilmann KP, Beekmann SE, Miller NJ, Miller AL, Rice CL, et al. Macrolide-resistant *Streptococcus pyogenes* in the United States, 2002-2003. Clin Infect Dis. 2005;41:599–608. 10.1086/432473 16080080

[15] Lancefield RC. The antigenic complex of *Streptococcus haemolyticus*: I. Demonstration of a type-specific substance in extracts of *Streptococcus haemolyticus.* J Exp Med. 1928;47:91–103. 10.1084/jem.47.1.91 19869404 PMC2131344

[16] Turner CE, Holden MTG, Blane B, Horner C, Peacock SJ, Sriskandan S. The emergence of successful *Streptococcus pyogenes* lineages through convergent pathways of capsule loss and recombination directing high toxin expression. MBio. 2019;10:e02521–19. 10.1128/mBio.02521-19 31822586 PMC6904876

[17] Wattam AR, Abraham D, Dalay O, Disz TL, Driscoll T, Gabbard JL, et al. PATRIC, the bacterial bioinformatics database and analysis resource. Nucleic Acids Res. 2014;42(D1):D581–91. 10.1093/nar/gkt1099 24225323 PMC3965095

[18] Sullivan MJ, Petty NK, Beatson SA. Easyfig: a genome comparison visualizer. Bioinformatics. 2011;27:1009–10. 10.1093/bioinformatics/btr039 21278367 PMC3065679

[19] Nir-Paz R, Korenman Z, Ron M, Michael-Gayego A, Cohen-Poradosu R, Valinsky L, et al. *Streptococcus pyogenes emm* and T types within a decade, 1996-2005: implications for epidemiology and future vaccines. Epidemiol Infect. 2010;138:53–60. 10.1017/S0950268809002805 19480723

[20] Sanderson-Smith M, De Oliveira DM, Guglielmini J, McMillan DJ, Vu T, Holien JK, et al.; M Protein Study Group. A systematic and functional classification of Streptococcus pyogenes that serves as a new tool for molecular typing and vaccine development. J Infect Dis. 2014;210:1325–38. 10.1093/infdis/jiu260 24799598 PMC6083926

[21] Li Y, Rivers J, Mathis S, Li Z, Velusamy S, Nanduri SA, et al. Genomic surveillance of *Streptococcus pyogenes* strains causing invasive disease, United States, 2016–2017. Front Microbiol. 2020;11:1547. 10.3389/fmicb.2020.01547 32849323 PMC7396493

[22] Berbel D, Càmara J, García E, Tubau F, Guérin F, Giard JC, et al. A novel genomic island harbouring *lsa*(E) and *lnu*(B) genes and a defective prophage in a *Streptococcus pyogenes* isolate resistant to lincosamide, streptogramin A and pleuromutilin antibiotics. Int J Antimicrob Agents. 2019;54:647–51. 10.1016/j.ijantimicag.2019.08.019 31476434

[23] Seale AC, Davies MR, Anampiu K, Morpeth SC, Nyongesa S, Mwarumba S, et al. Invasive group A *Streptococcus* infection among children, rural Kenya. Emerg Infect Dis. 2016;22:224–32. 10.3201/eid2202.151358 26811918 PMC4734542

[24] Musser JM, Beres SB, Zhu L, Olsen RJ, Vuopio J, Hyyryläinen HL, et al. Reduced in vitro susceptibility of *Streptococcus pyogenes* to β-lactam antibiotics associated with mutations in the *pbp2x* gene is geographically widespread. J Clin Microbiol. 2020;58:e01993–19. 10.1128/JCM.01993-19 31996443 PMC7098749

[25] McGee L, Chochua S, Li Z, Mathis S, Rivers J, Metcalf B, et al. Multistate, population-based distributions of candidate vaccine targets, clonal complexes, and resistance features of invasive group B streptococci within the United States, 2015–2017. Clin Infect Dis. 2021;72:1004–13. 10.1093/cid/ciaa151 32060499 PMC8071603

[26] Gherardi G, Vitali LA, Creti R. Prevalent *emm* types among invasive GAS in Europe and North America since year 2000. Front Public Health. 2018;6:59. 10.3389/fpubh.2018.00059 29662874 PMC5890186

[27] Bessen DE, Carapetis JR, Beall B, Katz R, Hibble M, Currie BJ, et al. Contrasting molecular epidemiology of group A streptococci causing tropical and nontropical infections of the skin and throat. J Infect Dis. 2000;182:1109–16. 10.1086/315842 10979907

[28] Bessen DE. Tissue tropisms in group A Streptococcus: what virulence factors distinguish pharyngitis from impetigo strains? Curr Opin Infect Dis. 2016;29:295–303. 10.1097/QCO.0000000000000262 26895573 PMC5373551

[29] Jespersen MG, Lacey JA, Tong SYC, Davies MR. Global genomic epidemiology of *Streptococcus pyogenes.* Infect Genet Evol. 2020;86:104609. 10.1016/j.meegid.2020.104609 33147506

[30] Baroux N, D’Ortenzio E, Amédéo N, Baker C, Ali Alsuwayyid B, Dupont-Rouzeyrol M, et al. The *emm*-cluster typing system for Group A *Streptococcus* identifies epidemiologic similarities across the Pacific region. Clin Infect Dis. 2014;59:e84–92. 10.1093/cid/ciu490 24965347

