# Supplementary material for: Invasive Multidrug-Resistant emm93.0 Streptococcus pyogenes Strain Harboring a Novel Genomic Island, Israel, 2017–2019
Source: Emerg Infect Dis. 2022 Jan;28(1):118–26. doi: 10.3201/eid2801.210733 (PMC8714194; doi:10.3201/eid2801.210733)
Supplement: Appendix 2 — Additional figures for study of invasive multidrug-resistant group A Streptococcus pyogenes emm93.0 type outbreak, Israel, 2017–2019. [file 21-0733-Techapp-s2.pdf]

Article DOI: https://doi.org/10.3201/eid2801.210733

Invasive Multidrug-Resistant *emm*93.0 *Streptococcus pyogenes* Strain Harboring a Novel Genomic Island, Israel, 2017–2019

Appendix 2

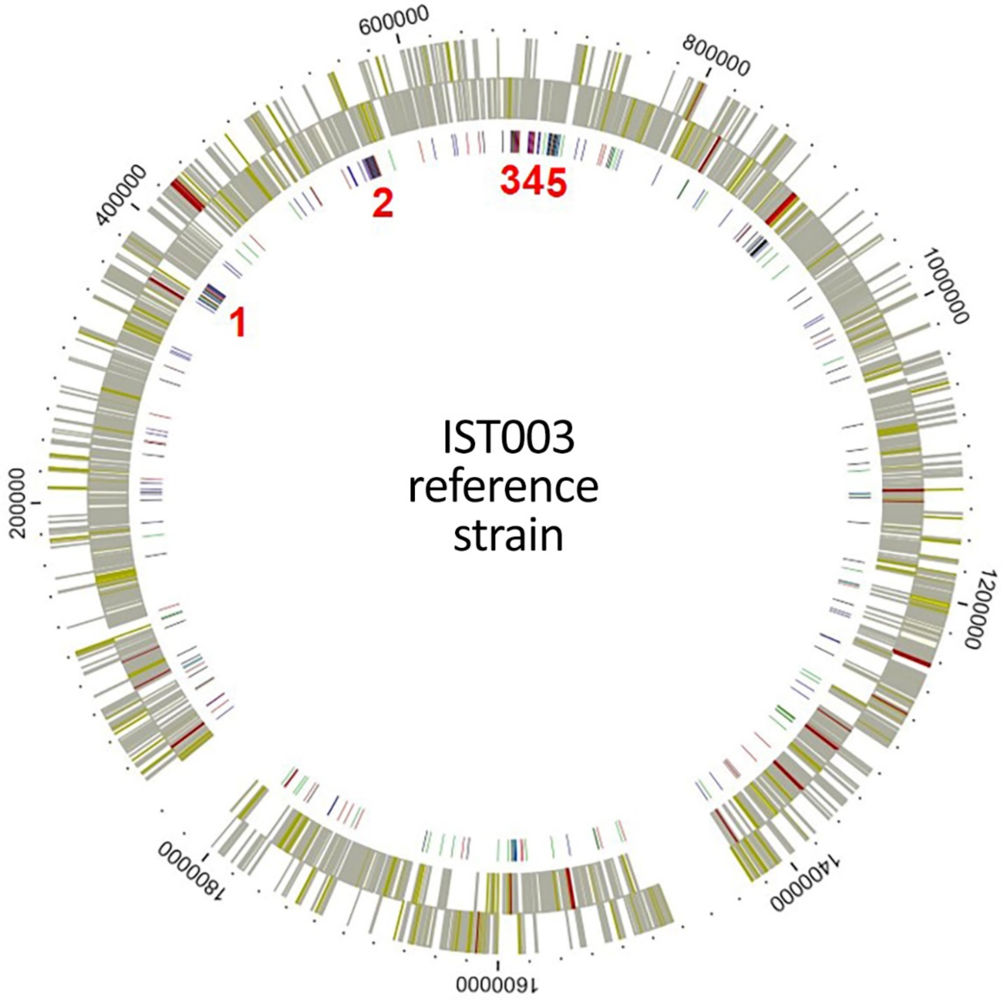

Appendix Figure 1. SNPs distribution in IST001 genome in comparison to the reference strain IST003. Inner cycle represents IST001 genome; colored lines represent SNPs in comparison to the reference strain. Outer cycles are CDS features of IST003. Numbers indicate the regions with high density of single nucleotide polymorphisms. Genes and locus tags identified in these regions are listed in Appendix Table 5 (https://wwwnc.cdc.gov/EID/article/28/1/21-0733-App1.xlsx).

Figure 8

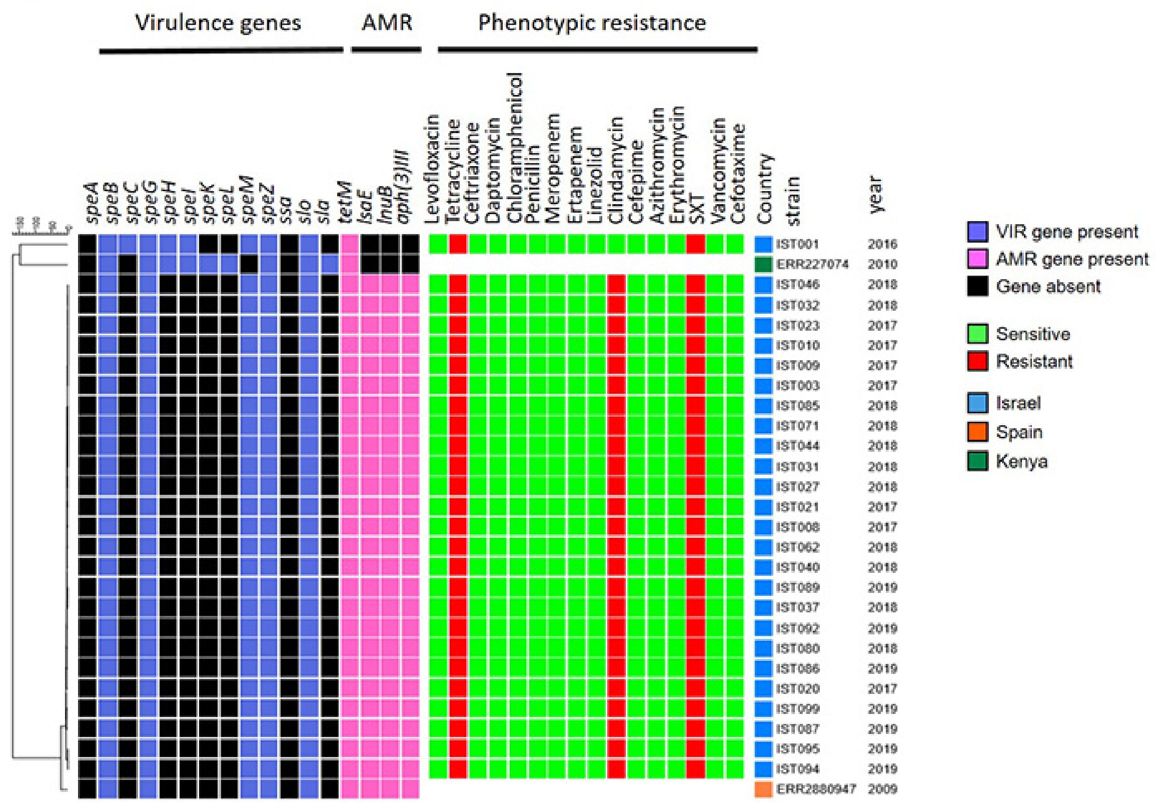

Appendix Figure 2. Presence of virulence and resistance genes among invasive group A *Streptococcus emm*93.0 type outbreak strains from Israel, previously identified strains from Spain and Kenya, and phenotypic resistance results. Each line represents a strain, presence or absence of SAg, and antimicrobial resistance genes. Characteristics are indicated by color. Antimicrobial agents tested are listed at the top of each column. The dendrogram indicates wgMLST allelic differences. Scale bars indicate substitutions per site. AMR, antimicrobial resistance; SXT, trimethoprim/sulfamethoxazole; VIR, virulence.
